# Perceptions of chemoprevention among individuals at high risk of oral cancer: qualitative study within the UK-based SAVER trial

**DOI:** 10.1136/bmjopen-2025-101326

**Published:** 2025-06-17

**Authors:** Frances Sherratt, Caroline McCarthy, Albert Jiménez-Tomàs, J Perry, R Kuruvilla, M W Ho, Stefano Fedele, M Daunt, S Moorhouse, R Shaw, Bridget Young

**Affiliations:** 1 Department of Psychological Sciences, University of Liverpool, Liverpool, UK; 2 University of Liverpool, Liverpool, UK; 3 Department of Pharmacology and Therapeutics, University of Liverpool, Liverpool, UK; 4 Oral and Maxillofacial Surgery, Leeds Dental Institute, Leeds, UK; 5 UCL Eastman Dental Institute, University College London, London, UK; 6 Department of Public Health, Policy and Systems, University of Liverpool, Liverpool, UK

**Keywords:** ONCOLOGY, PREVENTIVE MEDICINE, ORAL MEDICINE, QUALITATIVE RESEARCH, Clinical Trial

## Abstract

**Abstract:**

**Objectives:**

Clinical trials are needed to advance interventions such as chemoprevention that have potential to reduce the risk of malignant transformation in individuals with oral potentially malignant disorders. We explored the perspectives of those screened or invited to join an early phase clinical trial (the SAVER trial: Sodium valproate for the epigenetic reprogramming of high-risk oral epithelial dysplasia). Our objectives were to inform the SAVER trial while it was ongoing and to provide insights for future trials and chemoprevention therapy development more broadly.

**Design:**

Qualitative study involving audio-recorded, semistructured interviews. Analysis of transcribed interviews drew on thematic approaches.

**Setting:**

Five UK-based sites involved in SAVER.

**Participants:**

Purposive sample of individuals (n=20) with suspected or diagnosed oral epithelial dysplasia (OED) who were approached about SAVER.

**Results:**

Most interviewees readily accepted that OED warranted preventive treatment and were positive about the potential of chemoprevention. However, they were often concerned about the side effects of the trial medication, and together with a dislike of biopsies and a perception that the trial might disadvantage treatment, these concerns made some hesitant to participate in SAVER. Interviewees indicated that the communication of staff influenced their understanding and experience of the trial and identified several opportunities for enhancing these aspects.

**Conclusions:**

In indicating that individuals at risk of malignant transformation are accepting of chemoprevention in principle, our findings are supportive of future research on chemoprevention for this group. The findings also draw attention to the crucial role of communication in recruitment to chemoprevention trials. We provide recommendations to support staff during recruitment and enhance individuals’ experience of the trial.

**Trial registration number:**

ISRCTN12448611; Pre-results.

**STRENGTHS AND LIMITATIONS OF THIS STUDY**

- Our study was led by non-clinical researchers who were not closely involved in the SAVER trial but also involved clinicians who had key roles in SAVER, with the goal of providing balanced and contextualised insights on the trial.
- The sample was diverse in terms of age, sex, socioeconomic status, trial site and included individuals who consented, declined or were ineligible for SAVER, although we were unable to report the ethnicity of our sample.
- Interviewees were speaking of their perspectives within the context of a clinical trial, and the views of the wider population of those at risk of oral squamous cell carcinoma may be distinctive to the perspectives we report here.
- Well-publicised safety alerts about sodium valproate were issued just after our interviewing was completed, so the perspectives we report here may differ from the perspectives of individuals who were approached about SAVER following these alerts.

## Introduction

 Oral cancer, typically oral squamous cell carcinoma (OSCC), has high rates of morbidity and mortality, and its incidence is increasing.^1 2^ Oral potentially malignant disorders (OPMDs), many eventually harbouring oral epithelial dysplasia (OED), are a known risk factor for developing OSCC.^3^,^6^ Current management includes surgery and surveillance but neither treats the underlying condition, and both have other drawbacks: surgery is not always possible and can have high rates of recurrence, while surveillance often raises concerns about delays in detecting malignant transformation.^7^,^9^ There is a clinical need for effective interventions to reduce the risk of malignant transformation in individuals with OPMD, particularly for those diagnosed clinically and histologically with high-risk lesions. However, across a range of potentially malignant conditions, challenges have been reported in delivering chemoprevention trials of novel or repurposed therapeutics to reduce rates of malignant transformation,^10^ and while some trials have recruited successfully,^11 12^ others have encountered low uptake by eligible individuals.^13^,^15^ Compared with trials that investigate treatments for patients with an established diagnosis, chemoprevention trials generally have lower recruitment rates^16^ and encounter greater recruitment challenges.^17^,^19^ Such trials recruit individuals who are at risk, yet ostensibly ‘healthy’, and thus may perceive the burdens of these trials to be too great and the benefits too remote or uncertain.^20^ Understanding the perspectives of individuals at risk of cancer regarding chemoprevention trials is important to improve the acceptability of these trials and enhance communication and information resources for potential participants.

Much extant qualitative research exploring people’s perceptions of chemoprevention trials has been outside oncology. In line with the above suggestions regarding the perceived burden-benefit ratio of prevention trials,^20^ individuals at risk of rheumatoid arthritis have been found to doubt the need for treatment and be apprehensive about the risks of both chemoprevention treatment and trial participation.^18 21^ Relatively few studies have explored individuals’ perspectives on cancer chemoprevention trials. Qualitative research with postmenopausal women invited to join a chemoprevention trial for primary invasive breast cancer indicated that their deliberations about joining were complex and did not just involve weighing up the risks and benefits of the trial.^22^ Rather, the women’s decisions were also shaped by their experiences of breast cancer in relatives, their own previous investigations for breast cancer and their perceptions of taking chemoprevention medications and medicines in general.

As well as informing trials, awareness is also growing about the value of investigating stakeholders’ perspectives early in the development pipeline for new or repurposed chemopreventive agents.^23 24^ While chemoprevention has the potential to reduce the development of serious disease, this needs to be balanced with consideration of potential drawbacks, including the possibility of harm from overtreatment and increased healthcare costs without corresponding benefit.^25^ The importance of investigating the perspectives of potential recipients is also indicated by evidence that uptake and adherence to cancer chemoprevention among at-risk individuals is often poor when therapies are implemented in routine practice.^26^,^29^ Qualitative research examining women’s perspectives on breast cancer chemoprevention shows that they often regard taking preventive medication as burdensome, as a reminder of cancer risk and as a drug for people with cancer.^30^ Low uptake of chemoprevention for breast cancer has also been linked to problems in health professionals’ communication and to women’s concerns about medication side effects, complexities in their perceptions of cancer risks and scepticism about whether the risk of cancer onset can be reduced by primary prevention.^2831^,^35^

To our knowledge, no previous studies have explored perspectives of potential recipients of chemoprevention for OPMDs. As part of the SAVER trial (Sodium valproate for the epigenetic reprogramming of high-risk OED),^36^ we had an opportunity to start to address this gap. Specifically, we conducted a qualitative study, the SAVER Information Study, in which we interviewed individuals who were screened or invited to join the trial, to explore their perspectives on chemoprevention, their experiences of SAVER and how staff communicated about it. Multicentre chemoprevention trials have not been previously attempted in the UK for OPMDs, so SAVER raises questions about the trial’s acceptability to at-risk individuals and its feasibility. Thus, in addition to providing insights on how potential recipients view chemoprevention for OPMDs, the SAVER Information Study aimed to help inform communication and information giving about SAVER while the trial was running and to provide information to enhance the acceptability of any future phase III trial of sodium valproate for potential participants.

## Methods

### Design

**Figure 1:**
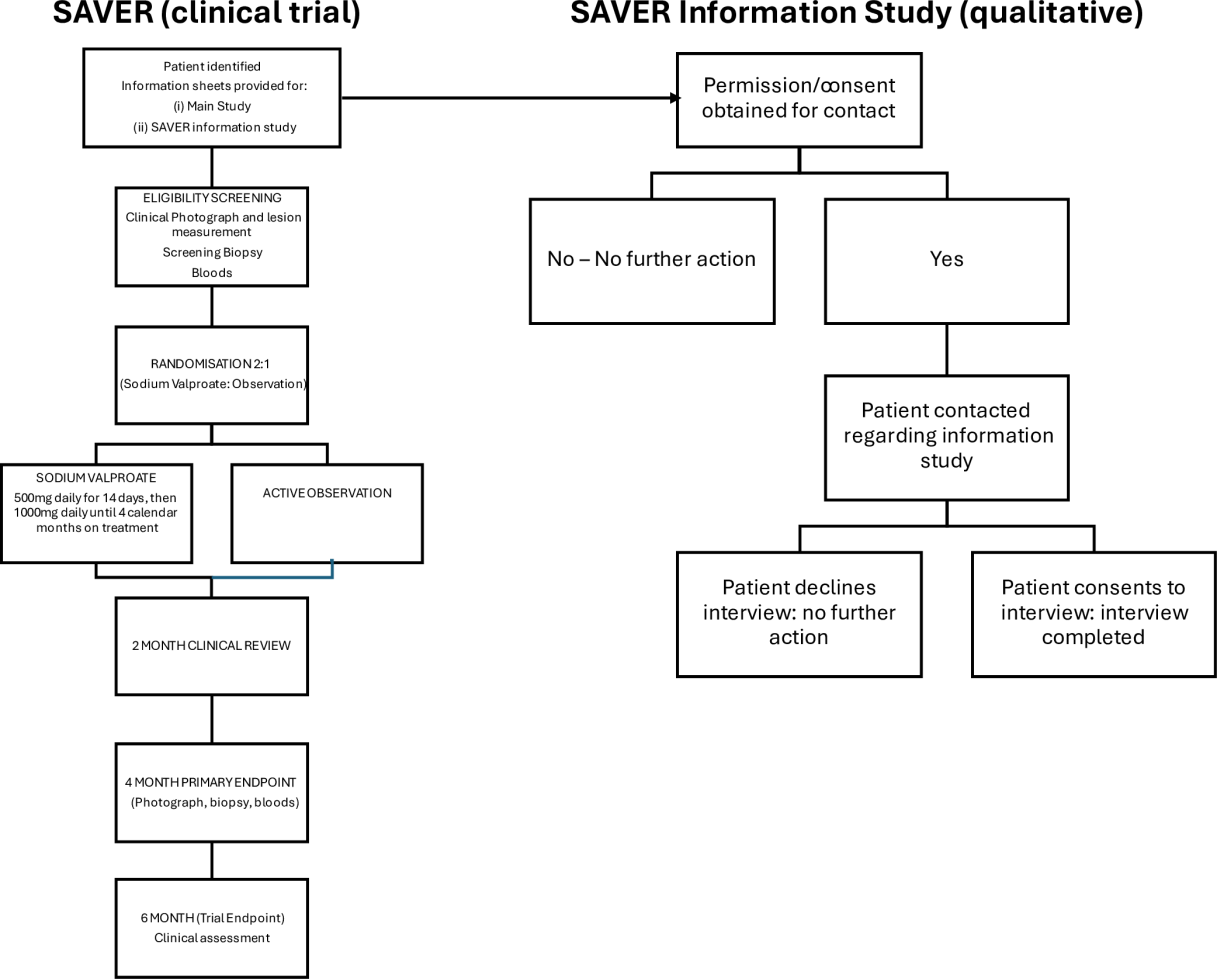
Overview of the design of SAVER and the SAVER Information Study.

Figure 1 summarises the design of the SAVER trial (ISRCTN12448611) and the qualitative Information Study. Briefly, SAVER was a phase IIB open-label, multicentre, UK-based randomised controlled trial of sodium valproate as a chemopreventive agent in individuals with high-risk OED and employing a window-of-opportunity design.^36^ SAVER’s primary aim was to gather preliminary evidence of the clinical and biological effects of valproate on OED (assessed via a composite surrogate endpoint comprising changes in changes in lesion size, heterozygosity profile and grade of OED) and to establish the feasibility and acceptability of a future definitive phase III trial. SAVER recruitment took place across 12 sites in the UK with a target of recruiting 110 participants. The trial closed in November 2024, at which point 80 participants had been randomised. Background information about changes to SAVER’s design and about sodium valproate can be found in box 1.

Box 1 **Background information about sodium valproate and changes to SAVER’s design**

**Sodium valproate**

Sodium valproate is a medication primarily used to treat epilepsy and bipolar disorder and to prevent migraines. Observational evidence suggests its use is associated with a lower incidence of head and neck malignancy.^56^ Valproate has been linked to a range of side effects at higher doses such as weight gain, tremor, drowsiness, cognitive slowing and, more rarely, liver or pancreatic disease. Valproate is also known to carry risks during pregnancy, significantly increasing the chance of birth defects. In 2015, the UK’s Medicines and Healthcare Products Regulatory Agency (MHRA) strengthened its warnings about the prescribing of valproate to females,^57^ and reflecting this, women of childbearing age have been ineligible for SAVER since the trial opened in October 2019. Subsequently, the MHRA issued further alerts in 2023 and 2024 stipulating that men and women under 55 years must not be prescribed valproate unless there is no alternative, and recommending the use of contraception for all males taking valproate where there is any risk of pregnancy.^44 45^ This latest guidance incorporating males arose from concerns about a greater frequency of neurodevelopmental disorders in the offspring of males prescribed valproate.

**Changes to SAVER’s design**

SAVER was originally designed as a double-blind placebo-controlled trial, with participants randomised 2:1 to a 4-month course of sodium valproate or to 4 months of matched placebo; after which, they return to their prior management pathway (surgery or surveillance). Recruitment at the first SAVER site started in October 2019 but was paused between March and July 2020 due to the COVID-19 pandemic. Challenges in securing the placebo necessitated a further recruitment pause in October 2020 while SAVER was redesigned. In September 2021, SAVER reopened as an unblinded RCT with an observation arm.

The SAVER Information Study entailed semi-structured qualitative interviews with individuals who had been approached about SAVER to explore their views and experiences of the trial. We were primarily concerned with understanding individuals’ experiences in a humanistic, interpretive approach^37^ while being sensitive to their needs and preferences.^38^ A UK National Research Ethics Committee (Ref. 18/NW/0180) approved SAVER and the SAVER Information Study.

### Patient and public involvement

Patients with experience of OED were involved throughout the study, providing advice on its design, the patient-facing materials, topic guide and interviews. Patients also contributed to the interpretation of the findings and to drafting the manuscript as co-authors.

### Participant recruitment

Clinical staff, which included oral and maxillofacial surgeons and consultants/specialists in oral medicine, approached individuals about the SAVER Information Study when obtaining consent for a SAVER screening biopsy. The screening biopsy was necessary to confirm a diagnosis of high-risk OED, which in part determined eligibility for SAVER. During this appointment, staff usually provided individuals with a participant information sheet detailing the Information Study. Prior to SAVER’s redesign, recruiters obtained verbal consent for the Information Study researcher to contact the individuals regarding an interview. Following the redesign, staff obtained written consent for the Information Study researchers (FS and RK) to contact individuals. Individuals were eligible for the Information Study if they had been screened or invited to join SAVER; there were no exclusion criteria. A purposive sampling approach aimed for diversity in interviewees’ age and sex, trial site, participation status and trial arm allocation. We drew on the concept of ‘information power’^39^ in deciding the adequacy of the sampling and resulting data. While the concept of data saturation is more common in qualitative research, information power moves the focus from the quantity of data collected to its quality, relevance and variability. It proposes that smaller samples are adequate if the data collected provide in-depth and varied insights about the phenomenon of interest.

### Procedure

The Information Study opened in six SAVER sites and recruited from October 2020 to April 2023. Researchers aimed to invite potential interviewees within approximately 1 or 2 months of their main discussion with a clinician about SAVER and to interview them several weeks later. Most interviews were conducted by FS, a qualitative researcher with a background in health research, while RK, a specialist registrar in clinical pharmacology and trained in qualitative interviewing, conducted two interviews. Following consent, audio-recorded semi-structured interviews were conducted by telephone. Interviews were topic guided (see online supplemental appendix A for an example version), with topic guides adapted in response to the ongoing study analysis and changes in SAVER’s design, as described above. Box 2 provides an overview of the topics explored during interviews. Interviewees did not receive compensation for their participation, as this was not a common practice when the Information Study was originally designed.

Box 2 **Example topics explored in interviews**

- Participant background.
- Introduction to SAVER.
- Views and perceptions of SAVER.
- Experience of being approached about SAVER.
- Views on interactions with health professionals involved in recruitment to SAVER.
- Key messages about SAVER.
- Views and understanding of trial arms.
- Views and understanding of randomisation.
- Reasons for consent or decline.
- Barriers or motivating factors to participating, including views on valproate.
- Views on written information about SAVER (eg, participant information sheet).
- General views on clinical research and prior experience of research involvement of interviewee or family member.
- Reflections on SAVER since being approached.

### Analysis

Audio-recorded interviews were transcribed, checked and pseudonymised. Analysis of transcripts was largely interpretive, examining not just the content of the interview but also how interviewees conveyed their narratives, including the words and phrases they used and what aspects they emphasised or de-emphasised. We worked both inductively and deductively, grounding the analysis in the data while also being guided by the research aims throughout. Analysis occurred at multiple levels, from detailed line-by-line coding to considering transcripts more holistically to contextualise the analysis. Procedurally, analysis drew on thematic approaches^40^ and involved: ‘familiarisation’ with the data, then generating categories and subcategories, followed by ‘indexing’ of the data according to these categories. BY, an experienced qualitative researcher, led the analysis, reading all transcripts multiple times and periodically discussing interpretations with CM, a clinical specialist in oral medicine who also reviewed and provided clinical insights on 17 transcripts. CM was involved in the clinical management of the remaining three interviewees and did not review their transcripts to protect their confidentiality. Additional input to the analysis was provided by FS. Several members of the SAVER Trial Management Group (TMG) reviewed a detailed initial report on the analysis containing extensive data extracts. Some SAVER TMG members also met with BY to identify lessons for enhancing communication and information resources about SAVER to improve the trial’s person-centredness and optimise recruitment. We referred to reporting guidance for qualitative research in writing this article.^41^

## Results

### Participants

FS and RK sent interview invitations to 49 individuals and conducted interviews with 20 individuals across five sites (seven interviewees were recruited from site A, two from site B, five from site C, three from site D and three from site E). Individuals who did not take part either did not respond to contact or declined due to time constraints or disinterest. Key characteristics of interviewees are listed in table 1.

**Table 1:** Interviewee characteristics

Interviewee	Age bracket	SAVER participation status	SAVER trial arm allocation
1	70+	Randomised	Blinded
2	30–49	Randomised	Blinded
3	30–49	Randomised	Blinded
4	50–59	Randomised	Unblinded (valproate)
5	60–69	Randomised	Unblinded (valproate)
6	70+	Randomised	Unblinded (observation)
7	70+	Ineligible	N/A
8	50–59	Randomised	Unblinded (valproate)
9	70+	Ineligible	N/A
10	50–59	Randomised	Unblinded (valproate)
11	70+	Ineligible	N/A
12	70+	Randomised	Unblinded (valproate)
13	60–69	Ineligible	N/A
14	70+	Declined	N/A
15	50–59	Declined	N/A
16	60–69	Declined	N/A
17	50–59	Declined	N/A
18	70+	Declined	N/A
19	30–49	Randomised	Unblinded (observation)
20	50–59	Randomised	Unblinded (observation)

Age ranges are used to maintain interviewee confidentiality.

N/A, not applicable.

Interviewees’ median age was 64 years, and just over half were female (n=11/20). According to English Indices of Deprivation 2019 deciles, which provide an indication of socioeconomic status,^42^ five interviewees lived in the most deprived areas of England (1–3), 11 in areas of average deprivation (deciles 4–7) and four in the least deprived areas (deciles 8–10). Eleven interviewees had been randomised in SAVER, five declined and four had been screened and approached but were ineligible for SAVER, usually because the biopsy showed no evidence of high-risk OED. Of the 11 interviewees participating in SAVER, all had been taking part in the trial for several weeks by the time they were interviewed.

### Qualitative results

We present the findings organised around four main themes: interpersonal communication and information; need for treatment and SAVER’s rationale; influences on decision-making; and opportunities to enhance information provision and experience. We include brief illustrative quotes in the main text while longer illustrative quotes are presented in table 2. All quotes are accompanied by identifiers (eg, I1) that can be cross-referenced with interviewees’ characteristics in table 1.

**Table 2:** Illustrative quotes

Quote number	
	**Interpersonal communication and information**
Q1	‘They're interested in doing their job, they're interested in furthering knowledge on this.’ (I8)
Q2	There was no pressure to do it whatsoever. He said… ‘You can stop at any time through it. And you don’t have to give us an explanation. You can just say, I am not doing it anymore, and that is it.’ He said, ‘It won’t have any difference on the amount of time you are waiting to get it lasered…so don’t go worrying.’ (I3)
Q3	‘[The doctor] went straight into… ‘So have you… have you had any thoughts about this?’ Nothing about the tongue, about my health or anything, it was instantly ‘Have you ever thought about this trial?’ And I had… barely sat in the seat.’ (I5)
Q4	‘You’re not thinking straight… and you just can’t remember.’ (I19)
Q5	‘[They’re] asking me to make a decision when… the… average layman is scared stiff… when cancer is mentioned.’ (I5)
	**Need for treatment and SAVER’s rationale**
Q6	‘I have no symptoms, it’s like… what are you on about kind of thing.’ (I10)
Q7	‘To reduce the possibility of all cells showing first signs of dysplasia to develop into full-blown cancer.’ (I4)
Q8	‘It had been discovered that people taking the drug had lower oral cancer diagnosis.’ (I16)
Q9	*Interviewer*: ‘I wondered what your thoughts were [about] those treatments being allocated… them being randomly allocated?’*Interviewee*: ‘Well, that’s how I always thought trials took place anyway. It wasn’t anything unusual.’ (I2)
Q10	‘I realised that it could help people in the future, wasn’t going to help me, I knew that. I mean it probably wasn’t going to help me. But it would help other people in the future and they could prescribe this medicine.’ (I7)
	**Influences on decision-making**
Q11	‘The very first time well… my thoughts then were I was quite happy to go along with it’ (I18)
Q12	‘He told me about it and he said, ‘Would I be interested in taking part?’ And I said, ‘Of course I would, yes, no problem at all,’ if it stops someone in the future having to have this much surgery as I’ve had because it either slows down or stops the progress of the pre-cancerous cells, then it can only be a good thing.’ (I2)
Q13	‘At least they are looking at my bloods… I am getting… slightly better service.Because I'm being looked at in detail because I am part of this trial.’ (I5)
Q14	‘I thought it might… stop it from turning into cancer. Because I knew in the past it had … I was really keen… to go on it. I thought… this is the answer… because at that point I'd already had I think about ten or eleven quite significant surgeries.’ (I20)
Q15	‘I spoke to the dentist actually about the sodium valproate… he was sort of concerned… … it started to open a can of worms if you know what I mean?’ (I18)
Q16	‘The problem was with my line of work… I just couldn’t commit the time… or the timings because of the places and times they wanted to sort of see me and take a biopsy and all that kind of thing. Just don’t fit with… my work at all.’ (I15)
	**Perceptions of valproate**
Q17	‘At that time, because everything felt stable for me… I just thought am I taking something that’s going to make me ill basically’ (I16)
Q18	‘They said it might reverse these patches on my tongue… and at that point I thought well… I just can't imagine them disappearing having looked at them.’ (I17)
Q19	‘The side-effects were a lot less than what I'm having to deal with… So… the risk… wasn’t really a consideration.’ (I20)
Q20	‘If I'm on my feet… I’ve got to keep… sitting on my chair… my legs… feel like jelly.’ (I12)
Q21	‘[The research nurses] they’ve given me all their contact details, you know so any concerns, phone them… and I'm not going to phone and say look… I keep forgetting to take the drug or… I'm forgetting… my train of thought… or I'm not sleeping well… I just think it’s part and parcel of being on this… I just hope in another two months then we can hopefully start weaning off it and… get back to normal.’ (I5)
Q22	‘I've completely… forgotten simple things like did I eat this thing or not? … That’s really scaring me at my age… I'm thinking I should talk to somebody and they’ll let me know, you know, if it’s part of this pill or what.…because if this is causing the side-effect… I definitely will come off the pill.’ (I10)
	**Perceptions of biopsies**
Q23	‘I hate having biopsies, I really do… my mouths always sore for… they say two weeks, but it really isn’t, it’s a long time. It takes a long time for it to settle down.’ (I16)
Q24	‘Anything to do with your mouth is painful, because you’ve got to speak and eat and drink and it’s not like a tummy operation where you can rest it. So, swallowing, drinking, eating, talking, when you’ve had work done on your mouth it’s quite painful.’ (I1)
Q25	‘I didn’t fancy the biopsy, I will tell you that… but it is a couple of days where you are in pain and I can put up with that’ (I3).
	**Perceptions of negative effects on treatment for OED**
Q26	‘I was able to go back and then say well look… I was under the impression that if cancer was found again, I… I would still be given this drug…and they assured me ‘Look if we find cancer… forget this trial, we will treat you immediately for any cancer that’s found… you will only take part in this trial if we know it’s safe to do so’… And that reassurance I wasn’t given early on.’ (I5)
Q27	‘I still really… was in favour of having it removed… rather than it sitting there… because every time you go in… every time you clean your teeth and you look at your tongue you’ll be thinking oh I wonder if it’s getting worse now or… I think I'd be fretting daily.’ (I14)
Q28	‘My first reaction was… will (the trial) slow down treatment… is there anything I might lose out on?’ (I13)
Q29	‘A phone call can't really tell you very much… they can’t really look in my mouth can they?’ (I8)
	**Opportunities to enhance information provision and experience**
Q30	‘Maybe a little bit more contact because… it’s a lot to take in at once and try and understand it. So obviously there’s leaflets [but] people can… not read them properly, and be worrying. Whereas if there’s someone to talk to a bit more… that knows about it… it would be a bit more relief with understanding things’ (I19)
Q31	‘This is what’s going to happen… week 1 we’ll do this… or month 2 we’re going to do this.’ (I10)
Q32	‘Why they picked that drug… there is no explanation as to why you would use epilepsy drugs like that. It is a bit random.’ (I3)
Q33	‘Politely skipped over… about the side-effects… when I told my children when I came back… and they said what was the side-effects? And I thought I didn’t even ask about that… they didn’t tell me any side-effects… and then when I went again and I asked what are the side-effects… then they told me.’ (I10)
Q34	‘It costs me because I'm self-employed… it’s taking… three to three and a half hours out of my day plus costing… parking’ (I8)
Q35	‘They gave me what they called a diary. Which was however… a few sheets printed out from a spreadsheet… that could have been better designed… for example if… the expectation that the patient will start taking the medication on [the] 21st of October… rather than (me) having to fill in… more of it could have been completed in advance. (I4)
Q36	‘It would be nice to know that you had contributed even in a small way… if it slows down the progression towards cancerous cells… I think I would be quite frustrated if it all went away and then I never found out whether the trial had any benefits. I would like to know… even if it was negative, at least you tried. But it would be nice to have a conclusion.’ (I1)
Q37	‘Having done it for eighteen weeks and all this, I would like to be kept informed of what the progress is… whenever I've asked people about that, it’s all ‘oh well we don’t even get told’… my consultant he says he doesn’t get told because it would affect his decision-making.’ (I20)

OED, oral epithelial dysplasia.

### Interpersonal communication and information

Recalling when they had first heard about SAVER, some interviewees spoke of feeling surprised or ‘a little taken aback’ (I16) when health professionals initially mentioned the trial, while others were ‘intrigued’ (I13) or saw their clinical team’s research involvement and enthusiasm for SAVER as a sign of their professional commitment (Q1). Despite these different responses when SAVER was introduced, most interviewees described their experiences of communication with staff in largely positive terms, remarking on their ‘helpful, gentle, thoughtful’ (I6) and ‘friendly’ (I15) demeanour, and made favourable comments about the verbal explanations and information that staff gave about SAVER, ‘I think they got it right’ (I16). All interviewees understood that participation in SAVER was voluntary, and many emphasised the extensive efforts of staff to ensure their understanding of this (Q2).

However, some interviewees expressed less favourable views regarding communication. Three staff were present when SAVER was first discussed with I17, who felt uncomfortable ‘sitting in the dentist chair with the lights on my face’, while a few interviewees remarked that some staff were more focused on recruiting to the trial than on patient care (Q3). Two interviewees who had been diagnosed with OED relatively recently described struggling to take in information (Q4) and make decisions about the trial in the aftermath of hearing the diagnosis (Q5). Both had initially been confused about some key issues such as the distinction between OED and cancer or what monitoring and treatment, if any, would be available to individuals who did not take part in SAVER. In contrast, interviewees who had a longer history of OED typically commented that they had found information relatively easy to absorb: ‘I think I took it in ok’ (I6). They also believed they had a good understanding of what taking part in SAVER would involve in practical terms, although a range of interviewees referred to specific questions that had not been covered in the verbal explanation or written information about the trial, as described below (see *Opportunities to enhance information provision and participant experience*).

### Need for treatment and SAVER’s rationale

Except for one interviewee, who reported initially voicing some doubts to the clinical team about the need to treat OED (Q6), no interviewees described ever having doubts about the need to monitor or treat the condition. Most understood that the trial was investigating if valproate could help prevent or delay OED developing into cancer (Q7), although they were also aware that there was no guarantee it would do so. Several interviewees recalled being told about the previous research that had informed SAVER (Q8), but a few had been initially unclear about ‘why they were using epilepsy drugs’ (I2) in SAVER, and of those recalling the previous research, few seemed to know that the findings were not directly applicable to OED. Most interviewees had some knowledge of randomisation (Q9), or knew that if they joined SAVER, they would not necessarily receive valproate. They typically understood that the evidence generated by SAVER was for the benefit of future at-risk individuals and not necessarily to benefit themselves (Q10). However, several interviewees seemed unclear that as an early phase trial, considerable time and further research would be needed after SAVER to enable valproate to be approved as a treatment for OED. For example, I15 spoke of wanting to find out when valproate would become available ‘as a form of treatment’ if SAVER’s results were promising.

### Influences on decision-making

Recalling the consultation when they first heard about SAVER, interviewees typically described an immediate willingness to participate (Q11), although they often continued to ponder their decision, and several who initially felt inclined to join subsequently declined SAVER. Whether or not they took part in SAVER, many interviewees emphasised their belief that research is ‘a good thing’ (I4) and almost all pointed to altruistic motives for wishing to take part (Q12). A few spoke enthusiastically about the ‘compelling’ (I8) research question that SAVER was addressing or were ‘amazed’ to hear about ‘the possibility of a drug helping the condition’ (I6). Some interviewees also described potential personal benefits of taking part in SAVER. For example, I5 believed participation in SAVER was benefitting them personally by providing better access to monitoring and to clinicians with greater expertise (Q13). Some also hoped that taking valproate might help to stop their OED developing into cancer (Q14). Regardless of whether they took part in SAVER, many interviewees nevertheless shared common concerns about the trial and noted that these concerns were often amplified by family members, friends and, occasionally, even health professionals such as dentists (Q15). Common concerns included perceptions that participation in SAVER might affect an individual’s well-being or work (due to potential side effects of valproate), or would be inconvenient (eg, requiring travel for additional appointments (Q16)). However, interviewees’ most prominent concerns were (i) negative perceptions of valproate, (ii) the additional oral biopsy/biopsies required by the trial protocol and (iii) worries that participation in SAVER might delay or disadvantage their treatment. We therefore focus on these in the following sections.

#### Perceptions of valproate

A few interviewees noted their reluctance to take medicines in general ‘I don’t take a lot of medication if I can avoid it’ (I16), but specific concerns about valproate and its side effects were often their main reason for declining SAVER. I18 believed that ‘not knowing what the side effects would be’ was probably their main reason for declining SAVER, while I16 felt their health had been ‘stable’ in recent years and was reluctant to take a medicine that might disrupt this (Q17). Despite believing that pregnancy was not a relevant consideration for them personally, I17 became concerned on hearing a pregnancy test would be required before joining SAVER due to valproate’s teratogenic effects. I17 was the only interviewee to express these concerns and reasoned that the teratogenic effects meant valproate was a ‘strong’ drug to take ‘if I don’t actually need it.’ Somewhat in tension with this view of valproate as a strong drug, I17 also doubted that the drug could have the desired effect on OED (Q18).

While concerned about the side effects of valproate, other interviewees indicated that these concerns did not deter them from taking part in SAVER. I4 was reassured when told by a doctor that the drug had ‘very few side-effects’, adding that ‘it’s like [the doctor] was saying take an aspirin every day.’ I6 was initially concerned about weight gain, hair and memory loss but reasoned that all drugs had side effects and was reassured when staff mentioned that the dose used in SAVER was ‘minimal’. The side effects of valproate played little or no part in the decisions of other interviewees or their concerns were far outweighed by other considerations. Indeed, many expressed a preference to be allocated to the valproate arm of the trial. I20 was particularly hopeful of benefitting personally from valproate. With scar tissue from the numerous surgeries required when their OED had previously progressed to cancer, I20 was worried that their treatment options were running out (Q19).

Several interviewees randomised to the valproate arm of SAVER spoke in detail of their experiences with the medication. They expressed little concern about the effort required to take the medicine, and none commented that taking the medicine acted as a reminder of their cancer risk. Rather, their narratives focused on the medication side effects, with some interviewees noting uncharacteristic levels of forgetfulness and tiredness that began soon after they started the medication (Q20). I12’s tiredness lessened after they had been on treatment for a few days but worsened again when, as scheduled, the dose of valproate increased. Nevertheless, I12 was clear that these problems were tolerable and believed the tablets might be ‘working’, having noticed fewer ‘white spots’ since taking the medication. I5 attributed recent memory problems and tiredness to valproate and, as a result, had forgotten to take the valproate on three occasions. However, I5 was reluctant to inform the trial team of the side effects and hoped these would resolve once participation in SAVER was complete (Q21). In contrast, I10 was ‘really freaked out’ about the memory problems they were experiencing and intended to discuss these with the trial team. I10 had already visited a general practitioner about the difficulties and been referred for investigations. While I10 was not necessarily attributing their memory problems to valproate, they intended to stop taking the drug if it became apparent that valproate was causing the difficulties (Q22).

#### Perceptions of biopsies

SAVER required two tissue samples from each participant but minimised the need for research-only biopsies by using excess tissue from samples taken for routine clinical purposes where possible. While the requirement for biopsies seemed to play little or no part in the decisions of some interviewees, many spoke of their profound dislike of biopsies and of the intense pain and eating difficulties they experienced for days afterwards (Q23, Q24). Against this backdrop, interviewees often noted that the requirement to have additional biopsies weighed heavily on their decisions about the trial. As I18 commented on their decision to decline SAVER: ‘I don’t want another biopsy unless it… was really necessary’. Interviewees with a long history of OED and OSCC often framed their comments in the context of having undergone multiple surgeries and biopsies for these conditions over many years. But regardless of how long ago they were first diagnosed, interviewees sometimes noted that staff did not prepare them for ‘how bad it (the biopsy) was going to be… or prescribe painkillers’ (I3) and remarked that staff tended to underestimate the pain and disruption that biopsies caused. While these considerations weighed on I3’s mind when deliberating about the trial (Q25), they were among several interviewees who ultimately decided to take part in SAVER despite reservations about biopsies.

#### Perceptions of negative effects on treatment for oral epithelial dysplasia

SAVER was a ‘window of opportunity’ trial with participation lasting 4 months, and this raised concerns about the possibility that joining the trial could delay or disadvantage treatment. After initially being approached about SAVER, I5 recalled becoming concerned about how taking part in the trial would affect their treatment. They had later telephoned the trial team for reassurance but voiced frustration that this reassurance had not been forthcoming when initially approached about SAVER (Q26). I14, whose concerns about the trial’s impact on treatment were prominent in their decision to decline SAVER (Q27), repeatedly referred to a close relative who had died of cancer because treatment had come ‘too late’, as well as to their own treatment for OSCC several years ago. This interviewee’s understanding of SAVER seemed tenuous, and at times, I14 implied the trial was comparing surgery versus valproate for treating OED. In contrast, other interviewees who voiced concerns about SAVER’s potential impact on treatment (Q28) were willing to join SAVER after hearing from staff that participation would not delay definitive treatment, because waiting lists for routine treatment for OED were comparable to the amount of time participants would be in the trial. Nevertheless, I20, who had previously undergone numerous treatments for OED and OSCC, recalled having to first ‘get my head in the right place… [that] you’ve got to leave it really for 18 weeks’. For I8, the in-person monitoring during the trial was influential in their decision to join SAVER, but they also expressed lingering concerns about the risk of progression during this period and doubts about the value of the check-in phone calls that participants received 2 months into trial (Q29).

### Opportunities to enhance information provision and participant experience

The above sections point to the interviewees’ varying experiences of communication and information provision about SAVER and to ways that these might be improved in the future (summarised in box 3). Additionally, several interviewees spoke of ways that their wider experience of SAVER might be enhanced (also summarised in box 3).

Box 3 **Summary of ways to enhance experience and information provision about SAVER**

**Communication**

- Be person-centred, not trial-centred.
- Ensure training and support is provided for all staff who discuss the trial with potential participants.
- Individuals may not be expecting to discuss research during consultations with health professionals, and opening statements about the trial, or the context of its introduction, can overshadow subsequent communication. Ensure introductory communication about the trial and the setting and timing is appropriate for the individual concerned.
- Those recently diagnosed with oral epithelial dysplasia (OED) are often worried and can struggle to absorb information about clinical trials on top of everything else. They may need more time and support to understand the trial than individuals with a longer history of OED.
- Individuals appreciate staff being enthusiastic about research and emphasising that participation in any clinical trial is voluntary.
- Individuals value discussing details of the trial with staff much more than reading the written participant information (and some do not read these materials at all). Ensure that potential participants do not feel rushed during discussions and make it clear that staff have time to answer their questions.

**Information provision during recruitment to SAVER**

- Ensure the rationale for investigating sodium valproate as a potential treatment for OED is given prominence and clearly explained during consultations.
- Maintain equipoise during discussions by being clear that there is genuine uncertainty about whether valproate will be of benefit. Also, ensure individuals are aware that the trial is not aiming to benefit participants and that further research will be needed before it is known whether sodium valproate is effective.
- Perceptions of sodium valproate and its side effects are a key influence on individuals’ decisions about SAVER. Be transparent about side effects, while providing reassurance that these are well understood. Consider which terminology is accurate for describing the dose of valproate in SAVER.
- Provide reassurance that participation in SAVER will not delay or disadvantage any treatment needed.
- Be transparent about whether additional biopsies are required for the trial, as this can be an important factor in individuals’ decisions about the trial. Many experience pain and difficulties eating after biopsies—they appreciate staff being candid about this and ensuring access to adequate pain control during and after the procedure.

**During participation in SAVER**

- Be aware that participants may be experiencing side effects from the medication. Some may spontaneously voice questions about these, while others may need to be prompted before discussing their concerns about side effects.
- Prioritise participant convenience around appointments and collection of medication.

**Other considerations**

- Name sodium valproate in the written information materials provided at screening.
- Produce a timetable or chart showing the trial’s timeline, key appointments and other trial requirements for participants.
- Some participants wanted updates on the trial’s progress while it was running; almost all were keen to find out about the trial’s findings on its completion.

Interviewees valued the verbal communication with staff about SAVER more than the written information, although the verbal communication needed to be readily available and appropriately paced. For example, I19, who had recently been diagnosed with OED and had several misunderstandings of SAVER, remarked that his consultations tended to be brief and advised ensuring that staff members had the time needed to explain about SAVER and answer individuals’ questions (Q30). I10 volunteered that they did not ‘read a single sheet’ of the written information and emphasised the importance of staff members meeting with potential participants in-person and spending time going through the information: ‘because we’re dealing with our condition, as well as what you’re saying to us’. I10 added that a ‘timetable’ or chart setting out key appointments and events over the course of SAVER would also be useful for participants (Q31).

Other interviewees perceived gaps or limitations in the information about valproate and suggested ways these could be addressed. Noting that the written information for the biopsy component of SAVER did not mention the name of the trial medication, I17 felt it was ‘super important’ for its name to be included so that potential participants could look up information about the medication online if they wished. I3 felt the written information about SAVER could have been clearer about the rationale for investigating valproate (Q32). Pointing out that the trial team had indicated the dose of valproate in SAVER was ‘very low’, I5 felt the dose given to participants in SAVER was being downplayed and that it would be more accurate to call it a moderate dose, while I10 believed that the side effects of valproate were downplayed during their verbal discussions with staff about SAVER (Q33).

Reflecting their sense of having gone to some lengths to take part in SAVER, several interviewees offered suggestions on ways to make taking part more convenient and enhance participants’ overall experiences of SAVER. I8 commented on the time, expense and inconvenience of having to travel to the hospital to pick up the valproate (Q34), while several interviewees questioned why participants could not collect the medication from local pharmacies or suggested sending the trial medication to participants by post or courier. I4 felt there was room to improve the design of the diary that participants were given to record their medication (Q35), while almost all interviewees wanted to be informed of SAVER’s findings when the trial is completed (Q36) and a few were keen to receive updates about its progress while the trial was running (Q37).

## Discussion

To our knowledge, individuals’ perceptions of chemoprevention for OSCC have not previously been investigated, and this study offers insights that may be useful for future research in this area. We explored the views of individuals with suspected or confirmed OED in the context of SAVER, a phase II randomised trial which is gathering preliminary evidence on the clinical and biological effects of valproate on OED. Almost all those interviewed readily accepted that OED could warrant preventive treatment. Compared with some previous studies indicating that women at risk of breast cancer were often resistant to chemoprevention as a risk-reducing strategy,^32 43^ interviewees in the current study were largely open-minded or hopeful about the potential of chemoprevention for OSCC. Those with a longer history of OED, including some previously treated for OSCC, were especially enthusiastic about the prospect of chemoprevention, and some hoped such treatment would become available to them relatively soon if valproate’s effects on OED were promising. While interviewees were broadly supportive of future chemoprevention research for OSCC, our findings also indicate the need to ensure individuals invited to participate in early phase trials such as SAVER are clear that further research is needed before treatments can be considered for roll out into clinical practice and that the timescale for this is usually very lengthy.

Despite most of our interviewees being receptive to the principle of chemoprevention, they had apprehensions about the adverse effects of valproate, and these were a prominent reason for some declining SAVER. This mirrors previous findings indicating that the side effects of cancer chemoprevention therapies are often a key concern for potential recipients.^28^,^3034 43^ Interestingly, the teratogenic effects of valproate did not, for the most part, contribute to these apprehensions in our study. While interviewees were generally aware of these effects and the associated publicity, most did not consider the teratogenic effects to be personally relevant. This is likely due to the exclusion of women with childbearing potential from SAVER in line with Medicines and Healthcare Products Regulatory Agency (MHRA) safety guidance. Additionally, interviews were completed before the MHRA issued the directives extending to males^44 45^ and the resulting adverse publicity on prescribing of valproate. We acknowledge individuals’ concerns about the teratogenic effects of valproate will likely have increased since our interviews were conducted, with implications for their willingness to join SAVER.

Regarding other adverse effects, interviewees emphasised the importance of reassurance by staff that valproate’s long track record in treating other conditions meant its side effects were well understood. Being informed that the dose of valproate in SAVER was lower than that typically used for other conditions was also reassuring, but it was important for this information to be conveyed accurately. Our findings also point to the importance of support being readily available for participants while on trial, including information that might help them manage adverse effects of medications such as tiredness, memory problems and weight gain. Knowing that some of these effects may gradually ease while on treatment and likely resolve once the medication is stopped may help participants to adhere to the medicine. Furthermore, although it might be anticipated that valproate’s decades-long association with conditions such as epilepsy might be off-putting, this was not a concern for most interviewees. However, a few interviewees advised making explanations about the rationale for investigating valproate more prominent to avoid potential participants being confused by this.

SAVER participants were required to have two tissue samples, one to ascertain eligibility and another after 4 months on trial to ascertain treatment response, although the number of research-only biopsies was minimised by using excess tissue from diagnostic samples where possible. Previous surveys investigating the influence of research biopsies on participation in trials have mostly focused on phase 1 trials in advanced cancer, with about 25%–50% of those surveyed reporting a reluctance to undergo research biopsies.^46^ Interviewees in our study who declined SAVER said the need for biopsies contributed to their decision, although none said it was the main reason they declined. However, regardless of their decisions about SAVER, interviewees emphasised the importance of staff taking an empathic approach when communicating about biopsies, including acknowledging the pain and disruption individuals often experience afterwards and providing advice on managing these difficulties. The perception that joining SAVER could delay or disadvantage their treatment was a further concern for interviewees, and this also called for careful verbal and written explanation. However, even with such explanation, trial teams need to be mindful that some individuals may continue to have residual fears that their OED might become malignant during the trial and benefit from ongoing reassurance regarding this.

Aligning with previous research on participants’ experiences of clinical trials, our interviewees pointed to the importance of person-centredness in research. This included minimising the trial’s demands on participants’ time and reciprocating their contribution to research by keeping them informed of the research progress and findings.^47^,^49^ The person-centredness of interpersonal communication during recruitment was especially important—interviewees were finely attuned to signs that staff were prioritising their care and well-being, or in a few cases, to signs that staff were more focused on the trial. Person-centredness in trials also extends to tailoring communication to the needs of individuals,^50^ recognising that, as we found, newly referred patients may struggle to understand aspects of the trial and would benefit from more time and support to absorb information than those with a longer history of OED. Similarly, most individuals we interviewed valued verbal communication with staff over written information, but for some, the opportunity for appropriately paced verbal discussions about the trial with staff was particularly important in enabling their understanding. This is consistent with evidence indicating that extended discussions are the most reliable way of improving an individual’s understanding of research and that face-to-face interactions are especially helpful for those from socioeconomically disadvantaged and ethnic minority backgrounds.^51 52^ Training and support for all staff who communicate with individuals about a trial is likely to be helpful in addressing these issues. As several interviewees were in contact with their community dental practitioners around the time of recruitment to SAVER, for future OSCC chemoprevention trials, there may also be merit in trial teams liaising with the community dentists of potential participants. Providing community dentists with key facts about such trials might enable them to better support potential participants.

As well as informing the suggestions in box 3 to enhance information provision about SAVER and the experience of those approached to take part, we provided the trial team with a written report on the findings and held a webinar to discuss the findings and identify opportunities to enhance participant recruitment and experience of the trial.

### Strengths and limitations

A strength of our study is that interviews and data analysis were led by non-clinical researchers who were not closely involved in SAVER but also involved clinicians who had key roles in SAVER. This aimed to ensure we reached balanced and contextualised insights on the trial. Our sample size was typical for qualitative studies to enable the in-depth data and analysis needed for generating insights into individuals’ perspectives,^53^ and the sample was diverse in terms of age, sex, socioeconomic status and trial site. Additionally, as well as including SAVER participants, we interviewed individuals who had declined SAVER or were ineligible after screening. Although with limited numbers in each subgroup, our analysis was more holistic than specifically focused on comparing these subgroups. Data from each subgroup were nevertheless detailed and varied, and as others have noted,^54^ including the perspectives of individuals with differing pathways in relation to the phenomenon of interest enriched the analysis. Additionally, given that research on clinical trials in cancer settings has mostly focused on the perspectives of trial participants,^55^ our findings add to the small evidence base regarding the perspectives of non-participants.

A limitation is that we are unable to report the ethnicity of our sample as, inadvertently, ethical approval omitted permission for this in our study design. This study was conducted in the UK, and research is needed in other countries to examine the broader transferability of the findings. As well as helping to inform chemoprevention clinical trials for OSCC, the findings also provide insights about how those potentially at risk of cancers regard chemoprevention therapy in general. However, interviewees were speaking of their perspectives within the context of a clinical trial, and the views of the wider population of those at risk of OSCC may be distinctive to the perspectives we report here. One temporal limitation is that recent well-publicised MHRA safety alerts on valproate^44 45^ came just after the data collection period, and anecdotal reports from staff involved in SAVER suggest the recent alerts have affected the sentiment of those approached and recruitment to SAVER.

## Conclusions

We found that individuals potentially at risk of OSCC held largely favourable views about chemoprevention, although they were concerned about medication side effects. These concerns were particularly prominent among those who declined SAVER, with dislike of biopsies and fear that the trial might disadvantage treatment also contributing to hesitancy about trial participation. Our findings draw attention to the crucial role of interpersonal communication during recruitment and indicate ways that individuals’ experience of SAVER and communication about the trial could be enhanced. We also suggest steps that might help to support participants in the aftermath of biopsies and with medication adherence while on trial. The findings have wider implications for future chemoprevention trials for those at risk of OSCC, particularly in indicating that the individuals we interviewed were broadly accepting of chemoprevention in principle.

## Supplementary material

10.1136/bmjopen-2025-101326 online supplemental file 1

## Data Availability

Data are available upon reasonable request.
