# Supplementary material for: Perceptions of chemoprevention among individuals at high risk of oral cancer: qualitative study within the UK-based SAVER trial
Source: BMJ Open. 2025 Jun 17;15(6):e101326. doi: 10.1136/bmjopen-2025-101326 (PMC12182162; doi:10.1136/bmjopen-2025-101326)
Supplement: online supplemental file 1 [file bmjopen-15-6-s001.docx]

**Supplementary Materials**

**Appendix A. Example interview topic guide**

- Reiterate aims and procedure of interview. *How studies like SAVER can be a lot to understand, so we’re doing this information study to learn ways to improve how we explain research to patients {provide overview of topics we will discuss}*.
- Reassure re confidentiality.
- Check patient is happy to continue with interview.

**Patient background:**

1. Perhaps if you wouldn’t mind I could start by asking, how you are doing at the moment in terms of your health?

**General introduction to study:**

2. Can I ask when your last appointment at the hospital was? Was the SAVER study discussed at that visit?

3. If you wouldn’t mind, can I ask you to think back to when you first heard about the SAVER? Who was it that first mentioned the SAVER? Can you tell me a little bit about that discussion?

4. Can we go back a bit and talk about before that appointment when SAVER was first mentioned. So, when you came in to see {clinician’s name} did you have any inkling of what would be discussed?

5. When the idea of SAVER was first raised, what kind of thoughts went through your mind?

6. When you came out of that appointment, what was your understanding of what SAVER involved?

**Study discussion:**

7. Can you tell me a little bit about what happened when you went to the discussion? What were the main things discussed?

8. In your own words, what did {clinician’s name} explain about the SAVER study? Is there anything about that discussion that stood out to you in particular?

9. What was it like trying to take in the information about the SAVER study? Do you think you were able to take in more information that day? Was there anything you found a bit unclear or confusing?

10. Was there anything about that made you feel a bit surprised?

11. Is there anything about how the SAVER study was explained that could have been handled a bit differently?

12. How would you say the discussion went on the whole? How was {clinician’s name} in dealing with you that day?

13. Did you know {clinician’s name} before he/she approached you about the SAVER? *Nature/depth of the relationship*? *Have your impressions of him/her changed since the discussion? How?*

14. How do you think {clinician’s name} felt about the SAVER study? *Depending on response prompt regarding perceived recruiter opinions around SAVER, whether they should participate, and value of study.*

15. Did anyone (friend or relative) go with you for that appointment? Did they offer any opinions about SAVER either during the discussion or later*.*

16. Did you have any other discussions with any of the other members of the clinical team about the SAVER when you were trying to decide? What did those discussions add to your understanding of SAVER? Did different members of the clinical team cover different aspects?

**Study design:**

1. In your own words, can you tell me why you think the research is being done, what’s its purpose?

2. And from where you’re sitting now, can you tell me about what you understand the SAVER to involve? *Prompt on key features, understanding of study arms if appropriate. Prompt for longer term involvement e.g. follow-ups, biopsies.*

3. What did you think about the treatments being compared? *Treatment preference?* Did you have any questions about the treatments for {clinician’s name} during the discussion? What did you think about the different treatments after discussing them?

4. What’s your understanding about how it is decided what treatment is given? What did you think when {clinician’s name} told you about that? *Probe on randomisation.*

5. Did you think that over when you were making your decision? Can you tell me a little bit about that?

6. Did you feel {clinician’s name} was interested/had time to listen to what you thought about the SAVER study? Did you feel able to ask questions about the study? How did you feel {clinician’s name} dealt with your questions?

7. I know it can be hard to think of questions on the spot - did you find that after you came home you had questions that you wanted to ask? Were you able to speak to anyone about your questions – was there a number you could ring or did you have to wait till the next appointment? Or was there anything else you wondered about the SAVER study?

8. Were there any aspects of the SAVER study that were of concern to you? In what ways?

9. And looking at it from the other side, were there any ways in which you thought taking part in the SAVER study might be of benefit to you?

**Decision Making:**

10. Can you tell me a little bit about how you made your decision whether to go into the SAVER study or not? Did you think about it on the way home or was it when you got home and had a sit down?

11. In thinking about the decision about the SAVER study, what sort of things did you consider?

12. Which of those things would you say influenced your decision? *Prompt about study arms and whether views of those changed at any point.* Did you ask {clinician’s name} for their opinion on taking part? What did they say?

13. Was it something that you had to think about for long? Were there some aspects that you took time to think about?

14. Did you find it to be an easy or difficult decision to make? Can you tell me why?

15. Was there anything you found particularly helpful in making up your mind? Was there anything you found unhelpful?

16. Apart from the clinical team, did you discuss the SAVER study with any family or friends following the discussion? Can you tell me a little bit about that? *Other sources (e.g. Internet, patient charities).*

17. What or who would you say had the most influence on your decision? Can you tell me why?

18. Did you ever feel under pressure when you were trying to make up your mind? *Probe for explanation if necessary.*

19. Did you feel that this was your decision to make? How confident were you that you could say yes or no to the study and that no one would mind either way? *If SAVER participant, how do you think it might have felt if you did say no? What do you think would have happened if you had said no?*

20. Did you feel you had enough time to think things over properly?

21. Now that a little time has passed, how do you feel about the decision you made?

22. How did you feel about the amount of information you received, both written information and the discussions you had with the clinical team?

**The information sheet:**

23. {Show patient the SAVER study PIS} Did you receive one of these leaflets? Did you get a chance to look over it? What did you think of it?

24. Was there anything about the leaflet that you found unclear? Anything helpful?

25. Were there any questions that you had that weren’t covered in the information sheet?

26. Was the information sheet any help in making your mind up about the study? Did anyone else at home look at it?

27. Any suggestions to improve the information sheet at all?

**Views on research:**

28. Have you or anyone you know been asked to take part in medical research before? Could you tell me a bit about that?

29. What do you think about medical research in general?

30. Has your experience with the SAVER study influenced that at all? In what ways?

31. From your experience, if you were asked by another patient who was making a decision about SAVER, what would you advise them? *Is there anything you’d like them to know that you would have found helpful?*

32. And what would be your advice for a new member of the clinical team involved in explaining SAVER to patients? What things you think they should know or do to help patients?

33. If I can ask you now to look into the future, maybe two or three years time. If the SAVER study came into your mind, would there be things that you would be curious about? Would you want to know about the findings of the study?

**Demographics:**

34. Age, gender, consented to the SAVER study, randomisation group (if applicable), clinicians the patient discussed the study with, and postcode (socio-economic status).

**Is there anything else that you think is important to mention?**

**Thank you very much for your time, it has been really helpful.**
