# Supplementary material for: Perceptions of chemoprevention among individuals at high risk of oral cancer: qualitative study within the UK-based SAVER trial
Source: BMJ Open. 2025 Jun 17;15(6):e101326. doi: 10.1136/bmjopen-2025-101326 (PMC12182162; doi:10.1136/bmjopen-2025-101326)
Supplement: Supplementary file 2 [file bmjopen-2025-101326.reviewer_comments.pdf]

PEER REVIEW HISTORY

BMJ Open publishes all reviews undertaken for accepted manuscripts. Reviewers are asked to complete a checklist review form and are provided with free text boxes to elaborate on their assessment. These free text comments are reproduced below.

ARTICLE DETAILS

Title (Provisional)

Perceptions of chemoprevention among individuals at high risk of oral cancer: qualitative study within the UK-based SAVER trial

Authors

Sherratt, Frances; McCarthy, Caroline; Jiménez-Tomàs, Albert; Perry, J; Kuruvilla, R; Ho, MW; Fedele, Stefano; Daunt, M; Moorhouse, S; Shaw, R; Young, Bridget

---

VERSION 1 - REVIEW

**Reviewer**	**1**
**Name**	**Phillips, Kelly Anne**
**Affiliation**	**Peter MacCallum Canc Ctr**
**Date**	**29-Mar-2025**
**COI**	**None**

The manuscript describes the findings of a qualitative study of the perspectives of people approached to participate in a randomised trial of chemoprevention for oral epithelial dysplasia. The findings are potentially important in providing information that could assist with appropriate recruitment to this and future chemoprevention trials.

1) The design of SAVER was altered during the course of the study from a blinded placebo-controlled trial to an unblinded study with an observation arm. Did the study provide any information about the acceptability of these two different designs?

2) Can the authors provide more information on the timing of the interviews? The data in the last column of Table 1 suggests that some participants were interviewed before they had started on study treatment (“N/A”) while others had perhaps already started the allocated treatment (“Blinded” Unblinded”). If that was the case, how might that have affected the findings? Is it possible that those who knew they had been allocated to observation might have had different perceptions about joining the trial? If a key aim was to learn more about how trial recruitment might be optimised, would it have been more appropriate to interview participants before they knew their treatment allocation?

3) In the sample of 20 people interviewed, only 5 had declined the study, can the authors comment on whether this was adequate? Given that saturation is the prevailing concept in sample size for qualitative studies, and the concept of information power likely to be less familiar to nonexpert readers, can the authors provide a brief description of this in the manuscript?

Reviewer **2**

Name **Braun-Inglis, Christa**

Affiliation **University of Hawai'i Cancer Center, Translational and Clinical Research**

Date **07-Apr-2025**

COI **None**

This is an a very well written manuscript on an important topic of patient perception of chemoprevention. Appreciate the opportunity to review such a well written paper on an interesting topic. I only have 2 very minor critiques:

1. Recommendation is to state the aims of the SAVER Information Study more clearly. I think the summarized manor in which they are presented at the end of the introduction to do not seem to highlight the research questions adequately.

2. Page 10-qualatative results, there is a colon used "need for treatment and SAVER's rationale" instead of a semi-colon which confuses the reader as to what the four main themes are.

VERSION 1 - AUTHOR RESPONSE

Reviewer 1: comments for authors	Authors' response
The manuscript describes the findings of a qualitative study of the perspectives of people approached to participate in a randomised trial of chemoprevention for oral epithelial dysplasia. The findings are potentially important in providing information that could assist with appropriate recruitment to this and future chemoprevention trials.	Thank you for your helpful comments on our manuscript.

1) The design of SAVER was altered during the course of the study from a blinded placebo-controlled trial to an unblinded study with an observation arm. Did the study provide any information about the acceptability of these two different designs?	Data from the blinded phase of SAVER were limited to only three individuals, all of whom took part in the trial, and we do not have any data from individuals who declined SAVER during its blinded phase. In the unblinded phase we had more interviewees, and a mix of SAVER participants and non-participants. However, blinding was no longer part of the trial at this point and so it was not a pertinent issue to explore with interviewees. In view of these issues, we do not feel our study can address questions about the acceptability of the blinded versus unblinded trial design.
2) Can the authors provide more information on the timing of the interviews?<br><br>The data in the last column of Table 1 suggests that some participants were interviewed before they had started on study treatment (“N/A”) while others had perhaps already started the allocated treatment (“Blinded” Unblinded”). If that was the case, how might that have affected the findings?<br><br>Is it possible that those who knew they had been allocated to observation might have had different perceptions about joining the trial? If a key aim was to learn more about how trial recruitment might be optimised, would it have been more appropriate to interview participants before they knew their treatment allocation?	We agree with the reviewers that more information on the timing of the interviews would be helpful and have therefore added the following sentence on page 9, paragraph 1: *“Of the eleven individuals participating in SAVER, all had been taking part in the trial for several weeks by the time they were interviewed.”*<br><br>The entries in the last column of Table 1 have no relevance to the timing of the interviews. “N/A” was entered only for the 9 interviewees who did not take part in SAVER, as trial arm allocation was not applicable for these individuals. For the 11 interviewees who took part in SAVER the entries in this column indicate the trial arm allocation only – they have no relevance to when participants were interviewed relative to starting the study treatment.<br><br>Our aims were broader than optimising trial recruitment. In particular, we were keen to find out what could be done to enhance individuals’ experience of the trial pathway as a whole. If we had only interviewed participants before they knew their treatment allocation, we would have been unable to address this question.
3) In the sample of 20 people interviewed, only 5 had declined	We have revised the paragraph on strengths and limitations in the discussion to address this (please

the study, can the authors comment on whether this was adequate?	see page 19, paragraph 2). Specifically, this now states*: "Additionally, as well as including SAVER participants, we interviewed individuals who had declined SAVER or were ineligible after screening, although with limited numbers in each subgroup our analysis was more holistic than specifically focused on comparing these subgroups. Data from each subgroup were nevertheless detailed and varied, and as others have noted, [56] including the perspectives of individuals with differing pathways in relation to the phenomenon of interest enriched the analysis. Additionally, given that research on clinical trials in cancer settings has mostly focussed on the perspectives of trial participants, [57] our findings add to the small evidence-base regarding the perspectives of non-participants."*
Given that saturation is the prevailing concept in sample size for qualitative studies, and the concept of information power likely to be less familiar to nonexpert readers, can the authors provide a brief description of this in the manuscript?	We have added a brief description of information power in the participant recruitment section of the methods (page 6, paragraph 1). This states *"We drew on the concept of 'information power'[43], in deciding the adequacy of the sampling and resulting data. While the concept of data saturation is more common in qualitative research, information power moves the focus from the quantity of data collected to its quality, relevance and variability. It proposes that smaller samples are adequate if the data collected provide in-depth and varied insights about the phenomenon of interest."*
**Reviewer 2: comments for authors**	**Authors' response**
This is an a very well written manuscript on an important topic of patient perception of chemoprevention. Appreciate the opportunity to review such a well written paper on an interesting topic. I only have 2 very minor critiques:	Thank you for your encouraging comments.
1. Recommendation is to state the aims of the SAVER Information Study more clearly. I think the summarized manor in which they are presented at the end of the introduction to do not	We have revised several sentences in the paragraph at the end of the introduction in an effort to clarify our statement about the aims of the SAVER Information (please see page 5, first paragragh). This now states: *"To our knowledge, no previous studies have explored perspectives of potential recipients of chemoprevention for OPMDs. As part*

seem to highlight the research questions adequately.	*of the SAVER trial (Sodium valproate for the epigenetic reprogramming of high-risk OED) [36] we had an opportunity to start to address this gap. Specifically, we conducted a qualitative study, the SAVER Information Study, in which we interviewed individuals who were screened or invited to join the trial, to explore their perspectives on chemoprevention, their experiences of SAVER and how staff communicated about it. Multicentre chemoprevention trials have not been previously attempted in the UK for OPMDs, so SAVER raises questions about the trial's acceptability to at-risk individuals and its feasibility. Thus, in addition to providing insights on how potential recipients view chemoprevention for OPMDs, the SAVER Information study aimed to help inform communication and information giving about SAVER while the trial was running, and to provide information to enhance the acceptability of any future phase III trial of sodium valproate for potential participants."*
2. Page 10-qualatative results, there is a colon used "need for treatment and SAVER's rationale" instead of a semi-colon which confuses the reader as to what the four main themes are.	We have replaced the colon with a semi-colon (page 9, first paragraph).

We have indicated the revisions to our manuscript in track changes. In addition, we have included two new references numbered 56 and 57 (due to Endnote formatting these are not shown in track changes).

VERSION 2 - REVIEW

**Reviewer**	**1**
**Name**	**Phillips, Kelly Anne**
**Affiliation**	**Peter MacCallum Canc Ctr**
**Date**	**12-May-2025**
**COI**	

I thank the authors for carefully considering and responding to my questions and congratulate them on this important research.

**Reviewer**	**2**
**Name**	**Braun-Inglis, Christa**
**Affiliation**	**University of Hawai'i Cancer Center, Translational and Clinical Research**
**Date**	**12-May-2025**
**COI**	

This is a well written paper on important topic. Appreciate that authors interviewed both SAVER participants as well as those who declined to participate in the SAVER Study. Also, think analysis and discussion show the importance of interviews and qualitative research to better understand barriers and facilitator for recruitment and retention of all clinical trials.
